# Comparative Transcriptome Analysis of Gill Tissue in Response to Hypoxia in Silver Sillago (*Sillago sihama*)

**DOI:** 10.3390/ani10040628

**Published:** 2020-04-06

**Authors:** Wanida Saetan, Changxu Tian, Jiawang Yu, Xinghua Lin, Feixiang He, Yang Huang, Hongjuan Shi, Yulei Zhang, Guangli Li

**Affiliations:** 1 Fisheries College, Guangdong Ocean University, Zhanjiang 524088, China; wanidamnk62@gmail.com (W.S.); tiancx@gdou.edu.cn (C.T.); yjw9902122020@163.com (J.Y.); lxh_13414934257@163.com (X.L.); fly476806029@gmail.com (F.H.); zjouhy@126.com (Y.H.); shihongjuan1990@163.com (H.S.); yuleizhang88@163.com (Y.Z.); 2 Guangdong Research Center on Reproductive Control and Breeding Technology of Indigenous Valuable Fish Species, Zhanjiang 524088, China; 3 Marine Ecology and Aquaculture Environment of Zhanjiang, Zhanjiang 524088, China

**Keywords:** *Sillago sihama*, hypoxia stress response, gene expression, RNA-Seq

## Abstract

**Simple Summary:**

Silver sillago (*Sillago sihama*) is a marine fish species with a high economic value. *S. sihama* is poorly resistant to hypoxia. However, hypoxia stress-related genes and pathways in *S. sihama* remain unclear. In this study, we compared gill tissues of *S. sihama* between hypoxia and normoxia groups and detected differentially expressed genes under hypoxia stress. Two gene families, such as cytochrome P450 and glutathione S-transferase were associated with the function of metabolic process under the hypoxia stress. This study will expand our knowledge about the molecular mechanism of the transcriptome response to hypoxia stress in *S. sihama*.

**Abstract:**

Silver sillago (*Sillago sihama*) is a commercially important marine fish species in East Asia. In this study, we compared the transcriptome response to hypoxia stress in the gill tissue of *S. sihama*. The fish were divided into four groups, such as 1 h of hypoxia (hypoxia1h, DO = 1.5 ± 0.1 mg/L), 4 h of hypoxia (hypoxia4h, DO = 1.5 ± 0.1 mg/L), 4 h of reoxygen (reoxygen4h, DO = 8.0 ± 0.2 mg/L) after 4 h of hypoxia (DO = 1.5 mg/L), and normoxia or control (DO = 8.0 ± 0.2 mg/L) groups. Compared to the normoxia group, a total of 3550 genes were identified as differentially expressed genes (DEGs) (log_2_foldchange > 1 and padj < 0.05), including 1103, 1451 and 996 genes in hypoxia1h, hypoxia4h and reoxygen4h groups, respectively. Only 247 DEGs were differentially co-expressed in all treatment groups. According to Kyoto Encyclopedia of Genes and Genomes (KEGG) pathway analysis, DEGs were significantly enriched in steroid biosynthesis, biosynthesis of amino acids, glutathione metabolism and metabolism of xenobiotics by cytochrome P450, ferroptosis and drug metabolism—cytochrome P450 pathways. Of these, the cytochrome P450 (CYP) and glutathione S-transferase (GST) gene families were widely expressed. Our study represents the insights into the underlying molecular mechanisms of hypoxia stress.

## 1. Introduction

Hypoxia is one of the most significant stressors for most aquatic animals, which affects the development of aquaculture. Hypoxia refers to a dissolved oxygen (DO) level of less than 2.0 mg/L in the aquatic environment, which can be accelerated by several factors, such as human activities, water pollution, and intensive fish farming [1]. To adapt to the hypoxic environment, fish produce a range of adaptive physiological mechanisms, such as a rapid change in cell metabolism using ATP [2], regulation of respiratory function [3], floating head [4], and neurological, immune, and hormonal responses [5]. Severe hypoxia can even affect fish reproduction, survival, and cell metabolism [6]. The fish gill is the primary organ for physiological exchanges with the surrounding environment [7]. The fish gill plays a dominant role in aquatic gas exchange and is capable of extensive remodeling in response to changes in the DO level [8]. Many fish respond to hypoxia by increasing the functional surface area of their gill [4]. When the fish returns to normoxic water, the hypoxia-induced gill remodeling is reversed due to the embedding of gill lamellae [9].

The sillago silver (*Sillago sihama*) is a popular species of the family Sillaginidae [10]. *S. sihama* is a tropical shallow-water fish species, which is widely distributed in the Indian Ocean and along the coasts of China, Korea, and Japan. It is known as one of the commercially important fish species in East Asia [11,12]. The population of *S. sihama* has dramatically reduced due to overfishing and environmental pollution [12]. Therefore, it is necessary to increase the yield of this fish species by artificial breeding. However, it is difficult to breed *S. sihama* by artificial breeding programs due to its hypoxia sensitivity, which causes fish mortality, especially during the first week of the larval stage [13].

Next-generation sequencing (NGS) technology has been widely used to determine the gene expression levels in different organisms [14]. Based on Illumina sequencing technology, researchers have identified a number of hypoxia stress-related genes and signaling pathways in several aquatic organisms, such as schizothoracine fish (*Gymnocypris eckloni*) [15], hooded seal (*Cystophora cristata*) [16], blunt snout bream (*Megalobrama amblycephala*) [17], channel catfish (*Ictalurus punctatus*) [18], crucian carp (*Carassius auratus*) [19], Nile tilapia (*Oreochromis niloticus*) [20], zebrafish (*Danio rerio*) [21] and Atlantic salmon (*Salmo salar*) [22]. Although a number of hypoxia stress-related genes and pathways have been identified in fish, the molecular mechanisms related to hypoxia response vary in different species.

To date, there is scant information on hypoxia stress-related genes and no report on transcriptome response to hypoxia stress observed in *S. sihama*. Therefore, the study aimed to examine the transcriptome response to hypoxia stress in *S. sihama* using RNA-seq technology. The transcriptome profiles of the gill tissues between hypoxia and normoxia were compared and analyzed by detecting differentially expressed genes under hypoxia stress and the interactions of their pathways. This study will expand our knowledge about the molecular mechanism of the transcriptome response to hypoxia stress in *S. sihama*.

## 2. Materials and Methods

### 2.1. Ethical Statements

All experimental protocols were approved by the Animal Research and Ethics Committee of Guangdong Ocean University (201903003).

### 2.2. Fish and Hypoxia Experiment

*S. sihama* (13.40 ± 1.05 cm of total length and 14.57 ± 3.17 g of body weight) were obtained from Donghai Island, Guangdong, China. Before the experiment, 100 individuals were selected for hypoxia stress pre-experiment. According to pre-experiment, the average asphyxiation point was 1.0 mg/L and the floating head phenomenon was 1.5 mg/L. In addition, 200 individuals were collected and transferred to four 50 L tanks with a bio-filtered water recirculation system. After the acclimation at 25 ± 1 °C temperature, 8.0 mg/L dissolved oxygen (DO) (normoxia) and 29‰ salinity, the experiments were conducted in a bio-filtered water recirculation system for 1 month. During the acclimation period, the fish were fed with a commercial diet twice per day. The water quality was checked every day, and the dead animals and particles were removed at once.

Healthy fish were randomly transferred to four aquarium tanks (50 L) at a density of 50 fish per tank. Each tank has 40 L of seawater (25 ± 1 °C temperature and 29‰ salinity). The concentration of DO was measured each hour interval by JPB-607A dissolved oxygen meter (INESA Scientific Instrument Co. Ltd., Shanghai, China). The experimental fish were divided into four groups, including hypoxia for 0 h (normoxia, DO = 8.0 ± 0.2 mg/L), hypoxia for 1 h (hypoxia1h, DO = 1.5 ± 0.1 mg/L), hypoxia for 4 h (hypoxia4h, DO = 1.5 ± 0.1 mg/L) and normal oxygen recovered in 4 h after hypoxia4h (reoxygen4h, DO = 8.0 ± 0.2 mg/L) (Table S1). In hypoxia group, pure nitrogen was added to reduce the DO level in the tank of the hypoxia group from (8.0 ± 0.2) mg/L to (1.5 ± 0.1) mg/L and maintain the DO level (1.5 ± 0.1 mg/L) in the water. After 1 h and 4 h of hypoxia stress, ten floating fish were selected for sampling from hypoxia 1 h and hypoxia 4 h groups, respectively. From normoxia and reoxygen4h groups, 10 normal swimming fish per group were selected for sampling. All fish were anesthetized in a eugenol bath (1:1000). Fish gill samples from each group were taken, immediately frozen in liquid nitrogen and stored at −80 °C for further analysis.

### 2.3. RNA Extraction and Illumina Library Preparation

The total RNA of gill (n = 3 per group) from the four groups was extracted using TRIzol reagent (Life Technologies, Carlsbad, CA, USA) following the manufacturer’s instructions. The RNA purity was monitored by NanoPhotometer spectrophotometer (Nanodrop 2000c, Thermo Scientific, Wilmington, DE, USA). The RNA integrity was checked by ethidium bromide staining of 28S and 18S ribosomal bands on a 1.0% agarose gel.

A total of 3 μg RNA was prepared for each Illumina library sequence. Sequencing libraries were generated using the NEBNext^®^ Ultra™ RNA Library Prep Kit for Illumina^®^ (NEB, Carlsbad, CA, USA) following the manufacturer’s instructions. The library fragments were purified using the AMPure XP system (Beckman Coulter, Beverly, MA, USA) to select 250–300 bp complementary DNA (cDNA) fragments and each library quality was assessed by the Agilent Bioanalyzer 2100 system (Agilent Technologies, CA, USA). Then, 3 µL of USER Enzyme (NEB, USA) was used with size-selected, adaptor-ligated cDNA at 37 °C for 15 min, followed by 5 min at 95 °C before PCR. PCR was performed with Phusion High-Fidelity DNA polymerase, universal PCR primers and Index (X) Primer. Finally, PCR products were purified by the AMPure XP system and library quality was assessed by the Agilent Bioanalyzer 2100 system. All clean libraries of sequencing data were submitted to the NCBI Sequence Read Archive (SRA) database (Accession No.: SRR9673344 - SRR9673355).

### 2.4. Data Filtering, Reads Mapping and Differential Expression Analysis

Based on the *S. sihama* reference genome and gene model annotation files (unpublished data), the index and paired-end of clean reads were established by Hisat2 v2.0.5 (https://anaconda.org/biobuilds/hisat2). The total number of reads was mapped to each gene using featureCounts v1.5.0-p3. The fragments per kilobase of exon model per million reads mapped (FPKM) was used to estimate the expressed gene levels [23]. Raw data (raw reads) in fastq format were processed through in-house perl scripts. Clean data (clean reads) were obtained by removing reads containing adapter, poly-N and low-quality reads from raw data. The Q20, Q30, and GC-content of the clean data were calculated.

Differentially expressed genes (DEGs) between the normoxia and hypoxia treatments were identified using DESeq2 R package (version 1.16.1) [24]. DESeq2 provides statistical routines for determining differential expressions in digital gene expression data using a model based on the negative binomial distribution [25]. The threshold for identifying significant DEGs was set to adjusted *p*-value (padj) < 0.05 and |log_2_ fold change| > 1.0. DEGs were then subjected to enrichment analysis of Gene Ontology (GO) functions, KEGG pathways and those with *p* < 0.05 were considered significantly enriched.

### 2.5. Validation of DEGs by Quantitative Real-Time Polymerase Chain Reaction (qRT-PCR)

To verify the validity of the DEGs from RNA-seq data, 14 DEGs were selected randomly for examination by the qRT-PCR. The first-strand cDNA was synthesized using the PrimeScript™ RT reagent kit with gDNA Eraser (Takara, China) using 1 μg of total RNA.The reaction mixture 20 µL of each sample contained 10 µL 2X SYBR Green MasterMix reagent (Toyobo, Japan), 2 µL of 1:10 diluted cDNA with double-distilled water (DDW), 6.4 µL DDW, and 0.8 µL of each primer (10 μmol/L). The qRT-PCR primers were designed by Primer v6.0 as listed in Table S2. The PCR conditions were carried out as follows: initial denaturation at 95 °C for 5 min, followed by 32 cycles of 30 s denaturation at 94 °C, annealing at 60 °C for 30 s and extension at 72 °C for 20 s. To standardize gene expression values, the ribosomal protein L7 (*rpl7*) gene was used as a reference [26]. The relative expression levels of DEGs were analyzed using the 2^−ΔΔCT^ method [27]. The statistical analysis was performed using SPSS v19.0.

## 3. Results and Discussion

### 3.1. Illumina Sequencing Assembly

We constructed 12 cDNA libraries from four groups with triplicates (Table 1). The raw reads in each library ranged from 42,655,494 to 59,525,298. After removing and filtering adapter, poly-N and low quality reads, a total of 553,591,836 clean reads were obtained. The number of clean reads, including 146,129,138 reads in hypoxia1h group, 146,054,350 reads in hypoxia4h group, 138,170,114 reads in reoxygen4h group and 123,238,234 reads in normoxia group with Q20 (96.24%–97.23%) and Q30 (90.38%–92.80%) levels were obtained (Table 1).

### 3.2. Differential Gene Expression Analysis

**Figure 1:**
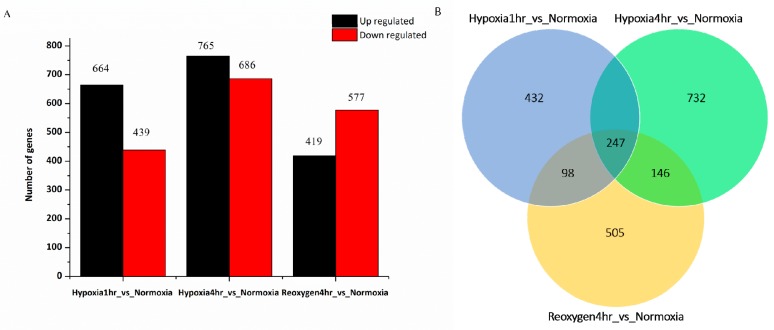
Different expression profiles. Notes: (**A**) The number of up- and down-regulated differentially expressed genes (DEGs) in each comparison group; (**B**) Venn diagram of all DEGs under hypoxia1h, hypoxia4h and reoxygen4h compared to normoxia group. Genes expressed only in hypoxia1h group (yellow circle); genes expressed only in hypoxia4h group (light blue circle); genes expressed only in reoxygen4h group (light purple circle). (log_2_foldchang > 1.0 and padj < 0.05).

A total of 3550 genes were identified as DEGs (Figure 1A). Compared to the normoxia group, 1,103 DEGs were detected in the hypoxia1h group, of which 664 DEGs were up-regulated and 439 DEGs were down-regulated. In the hypoxia4h group, a total of 1451 DEGs were identified, including 765 up-regulated and 686 down-regulated genes. In the reoxygen4h group, 996 DEGs were identified, including 419 up-regulated and 577 down-regulated genes (Figure 1A). Among these, the top ten up- and down-regulated annotated genes were presented in Table S3.

A Venn diagram analysis showed that 247 DEGs were co-expressed in all three treatment groups (hypoxia1h, hypoxia4h and reoxygen4h) (Figure 1B). Of these, 247 genes were co-expressed in all treatment groups, and insulin-like growth factor (*igfbp1*) and hypoxia-inducible factor prolyl hydroxylase (*egln3*) were down-regulated at reoxygen4h group but up-regulated in hypoxia1h and hypoxia4h groups (Table 2). A total of 432, 732, and 505 DEGs were expressed specifically in hypoxia1h, hypoxia4h and reoxygen4h groups, respectively, and 326, 146 and 98 genes were co-expressed in hypoxia1h and hypoxia4h, hypoxia4h and reoxygen4h, and reoxygen4h and hypoxia1h groups, respectively.

### 3.3. GO Term Enrichment Analysis

**Figure 2:**
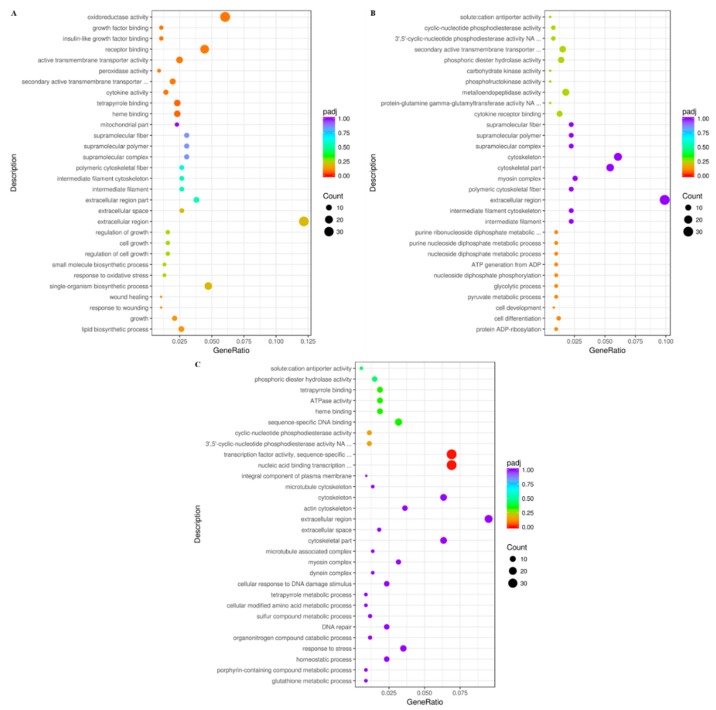
GO function classification of the differentially expressed genes (DEGs) comparison between the groups (padj < 0.05). (**A**) hypoxia1h_vs_normoxia, (**B**) hypoxia4h_vs_normoxia, (**C**) reoxygen4h_vs_normoxia. The *x*-axis represents the number of genes and the *y*-axis represents different Gene Ontology (GO) term functional classification.

In this study, all DEGs in each treatment group were enriched in GO and classified into three categories, including biological process (BP), cellular component (CC) and molecular function (MF). Two GO terms in MF categories were significantly enriched in the reoxygen4h group, including “nucleic acid-binding transcription factor activity” (GO:0001071) and “transcription factor activity, sequence-specific DNA binding” (GO:0003700) (Figure 2C). In contrast, no GO term was significantly enriched in the hypoxia1h and hypoxia4h groups (Figure 2A,B).

Compared to the normoxia group, the up-regulated DEGs were significantly enriched in 7 and 16 GO terms under hyoxia1h and the hyoxia4h groups, respectively (Table S4). However, no down-regulated DEG was significantly enriched in any GO under hypoxia1h and hyoxia4h groups (Table S4). In addition, down-regulated DEGs were significantly enriched in 4 GO terms under the reoxygen4h group (Table S4).

### 3.4. KEGG Pathway Enrichment Analysis

KEGG enrichment pathways were identified from DEGs. There were two KEGG pathways, including steroid biosynthesis pathway and biosynthesis of amino acid pathway, which were significantly enriched in the hypoxia1h group (Figure S1A). The steroid biosynthesis pathway was also significantly enriched in the hypoxia4h group (Figure S1B). In the reoxygen4h group, four enriched KEGG pathways were identified, including glutathione metabolism, metabolism of xenobiotics by cytochrome P450, drug metabolism cytochrome P450 and ferroptosis pathways (Figure S1C). All significantly enriched KEGG pathways and DEGs were shown in Table 3.

### 3.5. Validation of RNA-Seq Data with qRT-PCR

**Figure 3:**
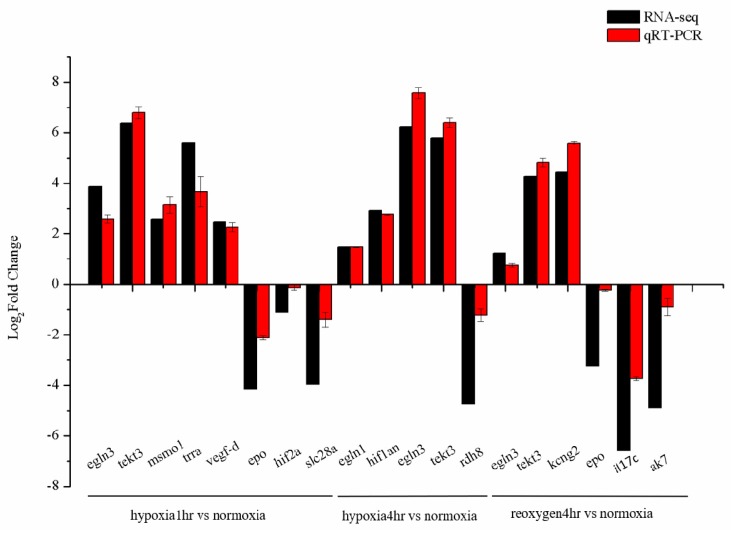
Comparison of gene expression data between RNA-seq and quantitative real-time PCR (qRT-PCR). The *x*-axis presents the gene name and the *y*-axis presents fold change in gene expression.

The results showed that the gene expression patterns of the two methods were consistent (Figure 3), indicating that the specific and accuracy of the transcriptome expression analysis. Among these, the fold induction values of *hif2a*, *rdh8* and *epo* genes showed significant differences between the treatment groups.

### 3.6. DEGs as Adaptive Response to Hypoxia

Globally, hypoxia is one of the major causes of economic loss in the aquaculture industry. In teleosts, gill is the first organ that responds to rapid changes in the DO level of water. Hypoxia disrupts the normal functions of the gill, including osmoregulation, respiration and nitrogenous waste excretion [28]. Therefore, the study about transcriptome response to hypoxia stress in gill tissue has significance.

In this study, we identified DEGs and their expression patterns under hypoxia stress in *S. sihama*. A previous study reported that the number of DEGs was increased with the increase of exposure time to hypoxia [29]. More DEGs up-regulated than down-regulated, which were also observed in other fish [29,30], indicated increasing gene expression levels as a major response for adaptation to hypoxic environments.

By comparing the three hypoxia treatment groups, genes involved in oxidative stress and immune response were significantly differentially expressed under hypoxia stress (Table 2). As a reported hypoxia-related gene in fish, *egln3* acts as a key factor to inhibit or stabilize hypoxia inducible factor alpha (HIF-1) under hypoxia [31]. *Igfbp-1* gene regulates the production of reactive oxygen species (ROS) in oxidative stress. Up-regulation of these genes were also observed in freshwater fish [15,32]. In addition, the *gimap4* and *gimap7* genes were significantly differentially expressed under hypoxia, and similar results were also observed in other fish [33,34]. The GTPase IMAP (gimaps) gene family regulate cell apoptosis and development and has a conserved function in the immune system of vertebrates [35,36], indicating that fish may respond positively to the hypoxic environments by regulating the immune response system mediated by the imaps gene family.

The CYP superfamily plays a critical role in the oxidative metabolism and the main enzyme is responsible for biosynthetic processes in the organism, such as steroid hormones, sterols and and vitamins [37,38]. In the present study, the up-regulated cytochrome P450 family 51 (*cyp51*) and cytochrome P450 family 24A1 (*cyp24a1*) genes were significantly enriched in the steroid biosynthesis pathway under hypoxia. The *cyp51* is a key factor of sterol biosynthetic pathways [39], which is regulated by cholesterol. This gene was involved in the reproduction and development in fish [40,41,42]. *Cyp24a1* plays a vital role in the metabolism and catabolism of vitamin D [43,44,45]. In this study, the up-regulated cytochrome P450 family 1A (*cyp1a*) gene was significantly enriched in the reoxygen4h group. The *cyp1a* gene family contains *cyp1a1* and cytochrome P450 family 1A2 (*cyp1a2*) subfamilies in mammals and other tetrapods [46]. Previous studies have reported that cyp1 displayed similar expression responses to environmental stress in aquatic animals [47,48]. The *cyp1* is involved in detoxifying the functions of environmental pollutants. Chronic intermittent hypoxia (CIH) decreased the expression of the hepatic *cyp1a2* gene in the drug metabolisom process of mouse [49].

The KEGG enrichment analysis showed that glutathione metabolism and metabolism of xenobiotics by CYPs were significantly enriched in the reoxygen4h group. The glutamate and glutamine genes, such as glutamate-cysteine ligase catalytic subunit (*gclc*), glutamate-cysteine ligase regulatory subunit (*gclm*), glutathione synthetase (*gss*) and glucose 6 phosphate 1 dehydrogenase (*g6pd*), were significantly differentially expressed in different treatment groups under hypoxic stress, indicating that the glutamate and glutamine gene families play an important role in transcriptional regulation of hypoxia.Glutathione maintains normal immune system function and has antioxidant effects. Among them, the *gclc*, *gclm* and *gss* were synthase and rate-limiting enzyme in glutathione. The main function of the *g6pd* gene is to produce NADPH, which is a key electron donor against oxidants. The *GST* superfamily is one of the major groups of enzymes widely expressed in organisms to protect cells from oxidative stress, cell imbalance and cell death [50,51]. Different aquatic animals showed diverse expression patterns of *gst* gene in response to hypoxia stress [52,53,54,55]. The results suggest that the regulation mechanism of glutathione metabolism in fish under hypoxic stress is complex and needs further study.

Ferroptosis is a common form of non-apoptotic regulated cell death, which was caused by different stimuli of stressor in animals [56]. Environmental stress leads to the occurrence of ferroptosis [21,50]. In this study, the ferroptosis pathway was significantly enriched in the reoxygen4h group. In this study, several up-regulated DEGs, such as glutathione genes (*gclm, gclc* and *gss*), transferrin (*tf*), ferritin heavy chain (*fth1*) and heme oxygenase 1 (*hmox1*) involved in the ferroptosis process were identified in the reoxygen4h group. Genes involved in ferroptosis pathway, such as *tf* and *gclc*, were also significantly increased under environmental stress in other fish [17,20]. It implies that the changes in environmental stress can lead to the damage of animal tissues, which has adverse effects on the exchange of oxygen and ion functions.

Several differentially expressed genes involved in the regulation of the glycolysis process were significantly expressed in the hypoxia1h group, such as pyruvate kinase (*pk*), phosphofructokinase (*pfk*), aldolase (*aldo*) and phosphoglycerate mutase (*pgam*) genes. The pk gene, a direct HIF-1 targeted gene, which was stimulated by the prolyl hydroxylase 3 (PHD3) [57] and involved in stress response of bacteria to hypoxia [58]. The *aldo* was involved in the mediated expression of HIF-1 gene under hypoxic stress [59]. The *pgam* gene was played a key role in the production of energy and biosynthesis of the organism, which is commonly expressed in the tumor cell [60]. Previous studies reported that the glycolysis process pathway was significantly enriched after exposure to the environmental stress in aquatic animals [61,62,63,64,65], indicating that this pathway plays an important role in participating in fish stress response.

## 4. Conclusions

This is the first transcriptome analysis to examine the gill response to hypoxia stress in *S. sihama.* The total number of up-regulated DEGs was found greater than the down-regulated DEGs with the increment of exposure time to hypoxia stress. In contrast, the number of down-regulated DEGs was found greater than up-regulated DEGs in the reoxygen treatment. Functional analysis of the DEGs showed that two GO terms and seven KEGG pathways were enriched. Our results provide a dataset to understand the regulatory mechanisms and molecular characteristics of hypoxia response in *S. sihama*.

## Figures and Tables

**Table 1:** Summary of gill transcriptome sequencing data.

Group	Raw Reads	Clean Reads	Clean Bases (G)	Q20 (%)	Q30 (%)	GC Content (%)
**Hypoxia1hr.**						
G_HI_1	59,525,298	57,377,574	8.61	97.00	92.30	47.58
G_HI_2	45,730,892	43,973,742	6.60	96.24	90.38	46.97
G_HI_3	48,080,268	44,777,822	6.72	96.87	92.04	46.98
**Hypoxia4hr.**						
G_HT_1	48,635,558	46,533,572	6.98	96.32	90.58	47.03
G_HT_2	49,539,404	47,922,836	7.19	96.94	92.18	47.52
G_HT_3	53,859,006	51,597,942	7.74	96.91	92.17	46.87
**Reoxygen4hr.**						
G_RO_1	47,007,630	44,659,448	6.70	96.59	91.37	46.63
G_RO_2	46,031,644	43,741,284	6.56	97.16	92.57	46.78
G_RO_3	52,813,132	49,769,382	7.47	96.58	91.37	47.32
**Normoxia**						
G_NO_1	42,655,494	40,115,434	6.02	97.23	92.8	47.31
G_NO_2	43,073,570	41,127,670	6.17	96.8	91.79	48.49
G_NO_3	44,331,564	41,995,130	6.30	96.75	91.75	47.69

**Table 2:** Annotation of the top ten differential expression genes in three treatment groups (padj < 0.05).

Gene Name	Log_2_ (Fold Change)			Description
	Hypoxia1h	Hypoxia4h	Reoxygen4h	
*gimap4*	6.39	6.45	6.89	GTPase IMAP family member 4
*tekt3*	6.39	5.79	4.27	Tektin-3
*hisat*	5.10	4.38	3.12	Histidine N-acetyltransferase
*kcnq2*	4.23	4.27	4.44	Potassium voltage-gated channel subfamily KQT r 2
*egln3*	3.86	6.21	−1.16	Hypoxia-inducible factor prolyl hydroxylase
*aqp9*	3.19	2.83	3.57	Aquaporin-9
*slc12a3*	3.12	1.67	−1.68	Solute carrier family 12 member 3
*igfbp1*	1.90	1.80	−1.17	Insulin-like growth factor-binding protein 1
*pde4*	1.36	1.89	−1.26	cAMP-specific phosphodiesterase 4
*pck1*	1.32	1.05	−1.06	Phosphoenolpyruvate carboxykinase (GTP)
*bmp10*	1.57	2.19	−1.88	bone morphogenetic protein 10
*trim16*	1.17	2.05	−1.31	Tripartite motif-containing protein 16
*kcnk1*	1.07	1.52	−1.32	Potassium channel subfamily K member 1
*cxcr4*	1.19	1.07	−1.69	C-X-C chemokine receptor type 4
*p4hb*	−6.47	−3.99	−6.60	Protein disulfide-isomerase
*tx_B*	−4.21	−4.59	−2.75	Tx beta-subunit
*glipr2*	−4.15	−1.58	−3.73	Golgi-associated plant pathogenesis-related protein 1
*gimap7*	−4.08	−4.95	−3.16	GTPase IMAP family member 7
*endod1*	−3.57	−3.61	−4.53	Endonuclease domain-containing 1 protein

**Table 3:** Significantly enriched Kyoto encyclopedia of genes and genomes (KEGG) pathways of differentially expressed genes (DEGs) in hypoxia1h_vs_normoxia, hypoxia4h_vs_normoxia and reoxygen4h_vs_normoxia group (padj < 0.05).

Pathway ID	Pathway Term	Gene Name
**Hypoxia1hr_vs_Normoxia**		
**dre00100**	Steroid biosynthesis	*meso1, cyp51, sqle, nsdhl, tm7sf2, fdft1, dhcr24, dhcr7, lss, ebp*
**dre01230**	Biosynthesis of amino acids	*pk, aldo, phgdh, pfk, aco, glnA, pgam, cps1, ass1, tktA, tktB, idh1 idh2*
**Hypoxia4hr_vs_Normoxia**		
**dre00100**	Steroid biosynthesis	*meso1, tm7sf2, sqle, dhcr24, lss, nsdhl, cyp51, fdft1, cyp24a1*
**Reoxygen4hr_vs_Normoxia**		
**dre00480**	Glutathione metabolism	*gclc, gsto1, gsr, LOC108897969, gss, gclm, gst, g6pd, LOC104939687, LOC104921330*
**dre00980**	Metabolism of xenobiotics by cytochrome P450	*gsto1, LOC108897969, cbr1, gst, cyp1a1, LOC104939687, ugt1a1*
**dre04216**	Ferroptosis	*gclc, fth1, gss, gclm, tf, hmox1, tfrc*
**dre00982**	Drug metabolism - cytochrome P450	*gsto1, LOC108897969, gst, LOC104939687, ugt1a1*

## Supplementary Materials

The following are available online at https://www.mdpi.com/2076-2615/10/4/628/s1, Figure S1: The KEGG pathway signification of differentially expressed genes (DEGs) comparison between groups. (A) hypoxia1h_vs_normoxia, (B) hypoxia4h_vs_normoxia, (C) reoxygen4h_vs_normoxia. The y-axis belongs to specific pathway, and the x-axis belongs to enrichment factor. The size and colors of the dots represent the number of genes and padj values, respectively (The dots with larger-size indicated a higher number of genes in the pathway). Table S1: The time points of experimental fish sampling under hypoxic stress and reoxygen condition. Table S2 Quantitative real time PCR (qRT-PCR) primer sequences data. Table S3: Annotation of top ten up- and down-regulated genes in the comparison between the groups in gill. (padj < 0.05). Table S4: Gene ontology (GO) of significantly genes up and down regulated differentially expressed genes (DEGs) comparison between treatment. Biological process (BP), cellular component (CC), and molecular function (MF), respectively.
