# Supplementary material for: Comparative Transcriptome Analysis of Gill Tissue in Response to Hypoxia in Silver Sillago (Sillago sihama)
Source: Animals (Basel). 2020 Apr 6;10(4):628. doi: 10.3390/ani10040628 (PMC7222756; doi:10.3390/ani10040628)
Supplement: Supplementary file 1 [file animals-10-00628-s001.pdf]

Supplementary Materials

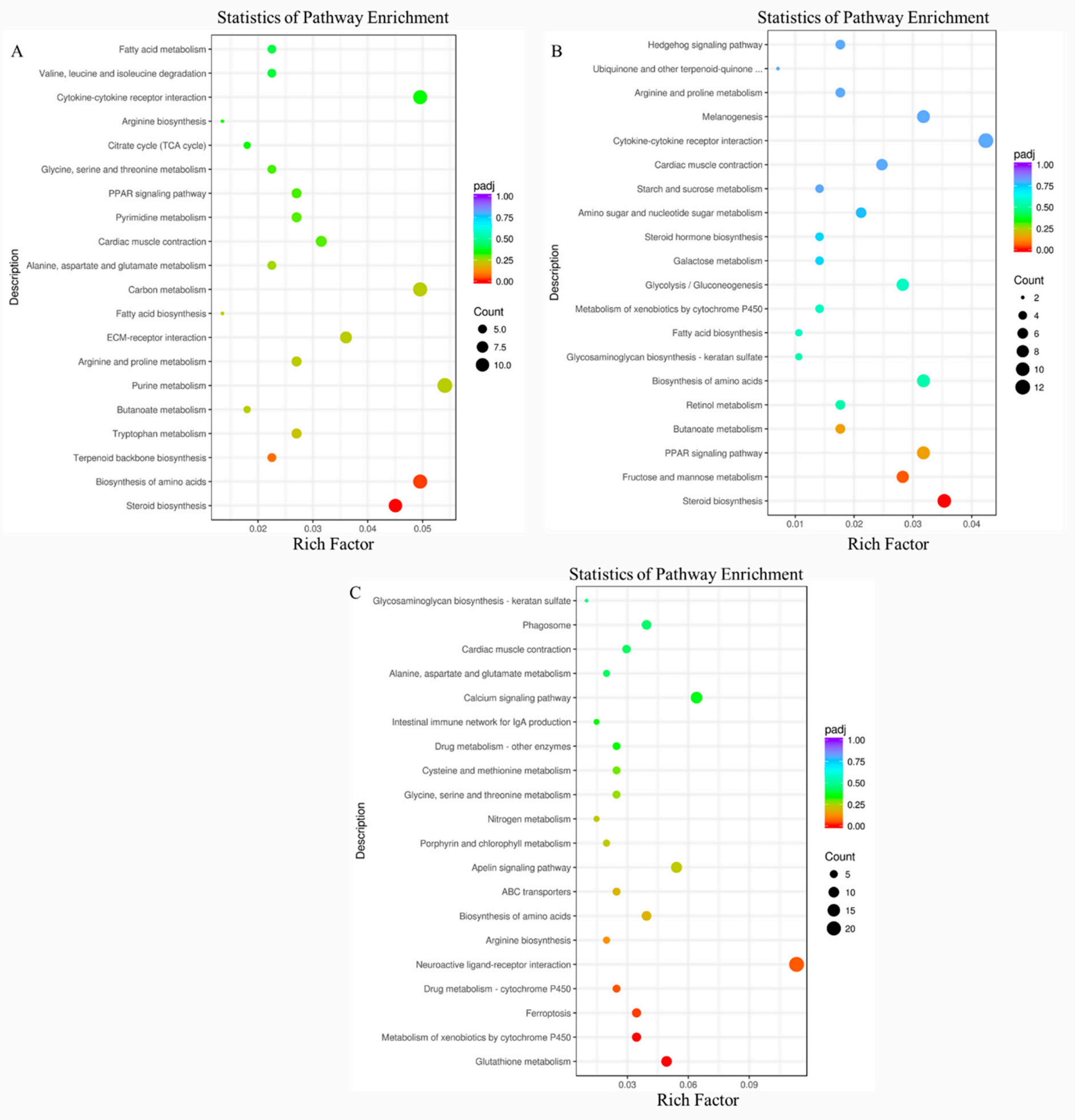

Figure S1. The KEGG pathway signification of differentially expressed genes (DEGs) comparison between groups. (**A**) hypoxia1h_vs_normoxia, (**B**) hypoxia4h_vs_normoxia, (**C**) reoxygen4h_vs_normoxia. The y-axis belongs to specific pathway, and the x-axis belongs to enrichment factor. The size and colors of the dots represent the number of genes and padj values, respectively (The dots with larger-size indicated a higher number of genes in the pathway).

Table S1. The time points of experimental fish sampling under hypoxic stress and reoxygen condition.

Time	Normoxia (mg/L)	Hypoxia1hr. (mg/L)	Hypoxia4hr. (mg/L)	Reoxygen4hr. (mg/L)
0 h	8.1	8.0	8.0	8.1
1 h	8.0	7.9	8.0	8.0
2 h	7.8	8.0	7.8	7.9
3 h	8.0	7.8	7.8	8.0
4 h	7.8	7.9	8.0	8.0
4.5 h	-	1.5	1.6	1.5
5.5 h	-	1.5	1.5	1.4
6.5 h	-	-	1.4	1.5
7.5 h	-	-	1.4	1.4
8.5 h	-	-	1.5	1.5
9 h	-	-	-	8.2
10 h	-	-	-	8.1
11 h	-	-	-	8.0
12 h	-	-	-	7.9
13 h	-	-	-	8.0

Table S2. Quantitative real time PCR (qRT-PCR) primer sequences data.

Gene name	Primer name	Sequence (5′-3′)
egln1	*egln1*-f	GTAGGTGCCGCAGCTCCTTCTA
	egln1-r	CGCTCTCCTCCGACTCTTGACT
hif1an	*hif1an*-f	CGCATTACGACGAGCAACAGAA
	hif1an-r	GCCGCCATTCAACAGTGATTCA
vegf_d	*vegf_d*-f	GCTGCTGAGGCTGACGGATT
	Vegf_d-r	TGTAAGATTCGGCGGCGTAGC
egln3	*egln3*-f	GCTGGAGCAGGTGAAGGAGATG
	egln3-r	TCGATGAGCGTGAGCAGGAAG
hif2a	*hif2a*-f	TCTGTGGAGAGCGACCTGAAGG
	hif2a-r	CTGGAAGAGGCTGGCGATGTTG
epo	*epo*-f	ACCGTCCGCCAGCAGATGAA
	epo-r	TCGTCACCAGCCAGGAAGCA
msmo1	*msmo1*-f	TGAACGGCACGGCAGACATCT
	msmo1-r	CCAGGCAGGCAGAAGAGGAAGT
tekt3	*tekt3*-f	GAGCCACGAGACCGACAACAT
	tekt3-r	ATGCCGCCTCATCCTCTCCT
trra	*trra*-f	TGATCCTGGTGAGCTGGACTGT
	trra-r	ATGCTGCGTGCCTGTCTCTG
slc28a3	*slc28a3*-f	GCAGAGACCAGATCCGCCTGAT
	slc28a3-r	GAGATGACCAGCAGCACCACAG
rdh8	*rdh8*-f	CGCCACCATGAGGAACCTGAGT
	rdh8-r	GCAACCGCACAAGCCCAAAGA
kcnq2	*kcnq2*-f	AGGAGGAGGAGAAGCAGGAGGA
	kcnq2-f	AGATGGAGAAGCCGCTGAAGGA
il17c	*il17c*-f	CGACGCCTACAGCCTCAACT
	il17c-r	CGACTGCCACGGACTTTAAGGT
ak7	*ak7*-f	AGACTGCCTGCTGCTGATGC
	ak7-r	TCCTGCTGCTGCTGCTTCAC
rpl7	*rpl7*-f	GCAAAGTGACCAGGAAACTGAT
	rpl7-r	GGCTGACACCGTTGATACCTCT

Table S3. Annotation of top ten up- and down-regulated genes in the comparison between the groups in gill. (padj < 0.05).

Gene names	log2FC	Description
**Hypoxia1hr_vs_Normoxia**		
gimap4	6.3933	GTPase IMAP family member 4
tekt3	6.3920	Tektin-3
taar	5.5958	Trace amine-associated receptor 1
taf11	5.5341	Transcription initiation factor TFIID subunit 11
hisat	5.1030	Histidine N-acetyltransferase
lyz	4.7917	Lysozyme C
erap1	4.7090	Endoplasmic reticulum aminopeptidase 1
hsp30	4.6502	Heat shock protein 30
kiss2	4.6355	Kisspeptin 2
fam163a	4.2682	Protein FAM163A
p4hb	-6.4724	Protein disulfide-isomerase
tpo	-4.3853	Thyroid peroxidase
il17a	-4.2462	Interleukin-17A
tx_B	-4.2110	Tx beta-subunit
glipr2	-4.1503	Golgi-associated plant pathogenesis-related protein 1
epx	-4.1489	Eosinophil peroxidase
pgrp	-4.1276	peptidoglycan recognition protein
slc28a3	-3.9583	Solute carrier family 28 member 3
p2rx4	-3.7564	P2X purinoceptor 4
zg16	-3.5903	Zymogen granule membrane protein 16
**Hypoxia4hr_vs_Normoxia**		
LOC107689127	7.9068	Uncharacterized
tm4sf5	7.1332	Transmembrane 4 L6 family member 5
apob	6.5043	Apolipoprotein B
gimap4	6.4504	GTPase IMAP family member 4
ddit4	6.2461	DNA-damage-inducible transcript 4
egln3	6.2118	Hypoxia-inducible factor prolyl hydroxylase
tekt3	5.7856	Tektin 3
mep1a	5.6665	Meprin A
chia	5.3216	Chitinase
ppp1r3g	5.1957	Protein phosphatase 1 regulatory subunit 3G
gimap7	-4.9539	GTPase IMAP family member 7
rdh8	-4.7320	Retinol dehydrogenase 8
tx_B	-4.5851	Tx beta-subunit
LOC103360465	-4.5562	Uncharacterized
fam69a	-4.1436	Protein FAM69A
p2rx4	-4.1092	P2X purinoceptor 4
p4hb	-3.9881	Protein disulfide-isomerase
slc28a3	-3.7257	Solute carrier family 28 member 3
plekhs1	-3.6995	Pleckstrin homology domain-containing family S member 1
endod1	-3.6091	Endonuclease domain-containing 1 protein
**Reoxygen4hr_vs_Normoxia**		
gimap4	6.8875	GTPase IMAP family member 4
ccdc183	4.7656	Coiled-coil domain-containing protein 183
kcnq2	4.4377	Potassium voltage-gated channel subfamily KQT member 2
gabrb2	4.3031	Gamma-aminobutyric acid receptor subunit beta-2
tekt3	4.2677	Tektin 3

mep1b	3.3933	Meprin B
gpt2	3.2669	Alanine aminotransferase 2
rnf183	3.2537	E3 ubiquitin-protein ligase rnf152-B
LOC106676196	3.2399	Uncharacterized
kiss2	3.1971	Kisspeptin 2
p4hb	-6.6011	Protein disulfide-isomerase
il17c	-6.5855	Interleukin 17C
ak7	-4.8801	Adenylate kinase 7
endod1	-4.5261	Endonuclease domain-containing 1 protein
cldnd	-4.5010	Claudin
cers2	-3.9373	Ceramide synthase 2
nr4a1	-3.9086	Nuclear receptor subfamily 4 group A member 1
unc13b	-3.8443	Protein unc-13 homolog B
glipr2	-3.7298	Golgi-associated plant pathogenesis-related protein 1
mfn1	-3.6066	Melanin-concentrating hormone receptor 1

Table S4. Gene ontology (GO) of significantly genes up and down regulated differentially expressed genes (DEGs) comparison between treatment. Biological process (BP), cellular component (CC), and molecular function (MF), respectively.

Expression	Category	GO ID	padj	Description
**Hypoxia1hr**				
Up-regulated	BP	GO:0008610	3.1E-02	Lipid biosynthetic process
	BP	GO:0009611	3.1E-02	Response to wounding
	BP	GO:0042060	3.1E-02	Wound healing
	BP	GO:0044711	4.5E-02	Single-organism biosynthetic process
	CC	GO:0005615	3.1E-02	Extracellular space
	CC	GO:0005576	4.1E-02	Extracellular region
	CC	GO:0044421	4.3E-02	Extracellular region part
**Hypoxia4hr**				
Up-regulated	BP	GO:0006091	9.7E-03	Generation of precursor metabolites and energy
	BP	GO:0006090	9.7E-03	Pyruvate metabolic process
	BP	GO:0006096	9.7E-03	Glycolytic process
	BP	GO:0006165	9.7E-03	Nucleoside diphosphate phosphorylation
	BP	GO:0006757	9.7E-03	ATP generation from ADP
	BP	GO:0009132	9.7E-03	Nucleoside diphosphate metabolic process
	BP	GO:0009135	9.7E-03	Purine nucleoside diphosphate metabolic process
	BP	GO:0009179	9.7E-03	Purine ribonucleoside diphosphate metabolic process
	BP	GO:0009185	9.7E-03	Ribonucleoside diphosphate metabolic process
	BP	GO:0016052	9.7E-03	Carbohydrate catabolic process
	BP	GO:0044724	9.7E-03	Single-organism carbohydrate catabolic process
	BP	GO:0046031	9.7E-03	ADP metabolic process
	BP	GO:0046939	9.7E-03	Nucleotide phosphorylation
	BP	GO:0044723	1.4E-02	Single-organism carbohydrate metabolic process
	BP	GO:0005975	1.4E-02	Carbohydrate metabolic process
	BP	GO:0044712	1.7E-02	Single-organism catabolic process
**Reoxygen4hr**				
Down-regulated	MF	GO:0001071	1.9E-08	Nucleic acid binding transcription factor activity
	MF	GO:0003700	1.9E-08	transcription factor activity, sequence-specific DNA binding
	MF	GO:0004114	9.9E-04	3',5'-cyclic-nucleotide phosphodiesterase activity
	MF	GO:0004112	1.1E-03	Cyclic-nucleotide phosphodiesterase activity
